# The Role of Female Reproductive Hormones in the Association between Migraine and Breast Cancer: An Unanswered Question

**DOI:** 10.3390/biomedicines11061613

**Published:** 2023-06-01

**Authors:** Paola Tiberio, Alessandro Viganò, Mariya Boyanova Ilieva, Sebastiano Pindilli, Anna Bianchi, Alberto Zambelli, Armando Santoro, Rita De Sanctis

**Affiliations:** 1 Medical Oncology and Hematology Unit, IRCCS Humanitas Research Hospital, 20089 Milan, Italy; paola.tiberio@cancercenter.humanitas.it (P.T.); mariya.ilieva@humanitas.it (M.B.I.); alberto.zambelli@hunimed.eu (A.Z.); armando.santoro@cancercenter.humanitas.it (A.S.); rita.de_sanctis@hunimed.eu (R.D.S.); 2 Neurology Unit, IRCCS Fondazione Don Carlo Gnocchi, 20148 Milan, Italy; abianchi@dongnocchi.it; 3 Department of Biomedical Sciences, Humanitas University, 20072 Milan, Italy; sebi.pindilli@yahoo.it

**Keywords:** breast cancer, migraine, headache, estrogens, aromatase, progestins, aura

## Abstract

Accumulating epidemiological studies have investigated a possible interconnection between migraine (Mi) and breast cancer (BC) because of the strong link between these diseases and female reproductive hormones. This review aims to consolidate findings from epidemiological studies and explore biologically plausible hypothetical mechanisms related to hormonal pathways. Current evidence suggests a protective role of Mi in BC development, particularly in case–control studies but not in cohort ones. The inconsistency among studies may be due to several reasons, including diagnostic criteria for Mi and the age gap between the development of these two diseases. Furthermore, recent research has challenged the concept of a net beneficial effect of Mi on BC, suggesting a more complex relationship between the two conditions. Many polymorphisms/mutations in hormone-related pathways are involved in at least one of the two conditions. The most promising evidence has emerged for a specific alteration in the estrogen receptor 1 gene (rs2228480). However, the possible specific mutation or polymorphism involved in this association has not yet been identified. Further studies with robust methodologies are needed to validate the protective role of Mi in BC and fully elucidate the precise nature of this causal relationship.

## 1. Introduction

Highlighting an actual clinical or pathophysiological association between two common diseases can be challenging because of the confounding effects of spurious overlaps. This is particularly true for highly prevalent medical conditions such as breast cancer (BC) and migraine (Mi). Both are predominantly gender-related disorders with a concomitant high prevalence in the global population. BC is the most common cancer in women [1], and Mi is the second most common form of headache worldwide, affecting about one in four women [2].

Therefore, when investigating the association between BC and Mi, the possibility that they may coexist in the same patient by pure chance is quite high. However, epidemiological data suggest an interplay between Mi and BC, indicating that one condition may influence the other. In particular, several studies have suggested that Mi might have a protective role in BC course. However, this has only been confirmed by a few studies, and several shortcomings may have influenced the different outcomes [3]. Another challenge is related to the age gap between the two conditions, as the Mi onset is generally years earlier than BC; therefore, by the time BC is diagnosed, Mi might be either resolved or forgotten.

**Figure 1:**
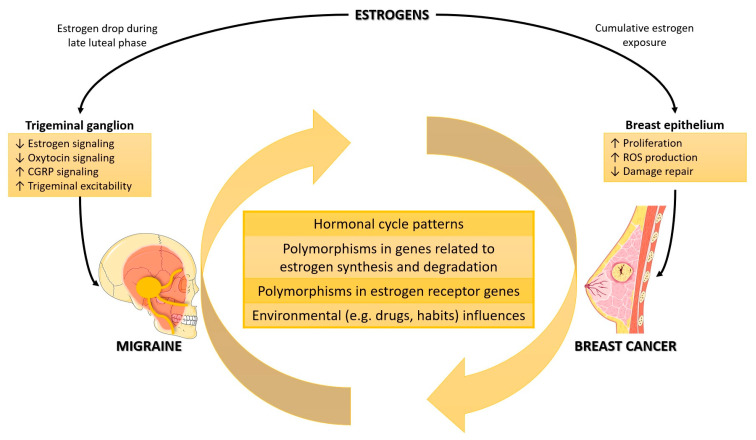
The association between estrogens, BC, and Mi.

This association seems to be explained by the activity of female reproductive hormones, which are strongly correlated with both BC and Mi (Figure 1). These hormones fluctuate within or outside a personal range over the lifespan. Approximately 75% of all BC express estrogen receptors (ER) and/or progesterone receptors (PR), commonly referred to as hormone receptors (HR) [4]. Hormone stimulation represents the main tumor progression driver in HR-positive disease [5]; therefore, targeted endocrine therapy is the mainstay of the treatment of this BC subtype. In addition, Mi is strongly related to female reproductive hormones, being more prevalent in women than in men and being more likely to occur during a specific time window of the menstrual cycle (−2 to +3 days of the menses [6]). In general, Mi attacks during menses are more severe, less responsive to acute treatments, and have a higher chance of relapse [7,8]. Furthermore, hormone replacement therapy has been linked to an increased risk of BC and Mi incidence (Table S1) [4,9].

Given the discrepancy in the available epidemiological data (which will be summarized below), the present review focuses on the role of female sex hormones in both BC and Mi and explores the possible overlapping mechanisms that may be involved in the association between these two disorders.

## 2. Epidemiological Data: Conflicting Evidence?

The first evidence of an interplay between BC and Mi dated back to 2008 and was first described in postmenopausal women [10], showing a reduced odds ratio (OR) of ductal carcinoma (OR = 0.67; 95% confidence interval [CI]: 0.54–0.82; *p* < 0.05) and lobular carcinoma (OR = 0.68; 95% CI: 0.52–0.90; *p* < 0.05) for Mi patients. One year later, the same Mi protective effect was also found in a pool of post- and pre-menopausal patients, with no difference between the two groups with an OR = 0.74 (95% CI: 0.66–0.82; *p* < 0.05) [11]. Interestingly, while the first study found that the clinical benefit was granted only to those patients who never used antimigraine treatments, the second study found an overall benefit not influenced by several confounding cofactors, including specific Mi therapies. An additional case–control study [12] supported these previous findings, pinpointing that BC patients had approximately half the likelihood of experiencing either Mi or tension-type headache (TTH) compared to the general population for both headache types.

On the negative side, these findings were only partially supported by two prospective studies conducted in 2013 and 2014. In the first study [13], authors did not find a significant association between Mi and BC overall and, by contrast, in sub-analyses differentiating among Mi patterns, an increased BC risk was shown in patients with a diagnosis of Mi without aura (MwoA) that was active at the moment of the study (hazard ratio = 1.21; 95% CI: 1.03–1.43; *p* < 0.05). A second prospective study [14], including 7696 Mi patients, of whom 3924 subsequently developed any type of BC, showed no association between Mi and (i) overall BC (hazard ratio = 0.96; 95% CI: 0.88–1.04; *p* = 0.28), (ii) in situ BC (hazard ratio = 0.97; 95% CI: 0.82–1.15; *p* = 0.74), or (iii) invasive BC (hazard ratio = 0.95; 95% CI: 0.87–1.04; *p* = 0.30) through a Cox proportional hazards approach. The second study presented data through a meta-analysis of previous investigations, which yielded results that differed from those of the observational study. Namely, the meta-analysis on about 268,215 patients found a 20% lower risk of overall BC occurrence (pool relative risk [RR] = 0.84; 95% CI: 0.73–0.98; *p* ≤ 0.001) [14]. However, no effect was found when the authors divided the six studies considered in the meta-analysis into case–control (in which the benefit was still preserved) and longitudinal studies (in which no beneficial role of Mi was reported).

Along these lines, a subsequent study did not find any positive or negative association (adjusted hazard ratio = 1.03; 95% CI: 0.89–1.21; *p* = 0.6747) in the overall analysis. However, it did reveal a positive association between the risk of BC and the annual number of visits for Mi, which became significant over four visits per year (hazard ratio = 2.00; 95% CI: 1.17–3.41; *p* = 0.0112). Additionally, the presence of Mi was associated with an increased number of BC-related examinations (i.e., mammography and breast echography), suggesting a potential overall link between the severe forms of the two diseases [15].

To summarize these findings, recent meta-analyses have pooled previous data. One meta-analysis released in 2015 reported the protective effect of Mi on BC in both the overall analysis (OR = 0.77; 95% CI: 0.64–0.92; *p* = 0.005) and the specific sub-analyses, including ductal or lobular histologies (OR = 0.84; 95% CI: 0.70–1.01; *p* = 0.060; and OR = 0.79; 95% CI: 0.70–0.90; *p* = 0.001, respectively), BC hormonal pattern (OR = 0.85; 95% CI: 0.77–0.94; *p* = 0.001), years from Mi onset (OR = 0.66; 95% CI: 0.48–0.90; *p* = 0.009; and OR = 0.68, 95% CI: 0.47–0.99, *p* = 0.042, respectively) but not patients’ age [16]. These findings were mostly confirmed via the second meta-analysis [17]. When examining the two studies mentioned above, it should be kept in mind that the strong similarity in their results is attributable to the fact that they both analyzed the same pool of six studies (Wu’s meta-analysis also included the prospective study conducted by Winter and coworkers, which was not considered in Rezaeian’s one).

The most recent meta-analysis by Hesari and colleagues [18] provided no further support for a pathophysiological association between MI and BC, as it confirmed the statistically significant negative correlation between Mi and BC in case–control studies but not in cohort ones. By stratifying for BC histological subtypes, the authors demonstrated a protective role of Mi for ductal and lobular carcinomas, with the latter exhibiting a more reduced risk than ductal cancers. Still, no correlation between Mi and BC was found when considering ER/PR status.

## 3. An Epidemiological Focus on the Role of HR

Several studies have attempted to deeply analyze the association between Mi and BC, considering the hormonal BC status. However, unclear evidence still emerged. In the study by Mathes, the authors showed that a history of Mi was associated with a reduced risk of postmenopausal BC, especially HR-positive BC subtypes [10]. These findings were later strengthened in a prospective study in 2010, as a sub-analysis of the Women’s Health Initiative Observational Study, which found the same results only for HR-positive BC patients while failing to find the same relationship for patients with an HR-negative profile in postmenopausal patients [19]. In addition, Lowry and colleagues demonstrated that a long personal history of Mi was a protective factor for HR-positive BC [20]. In the study by Ghorbani, which also included TTH patients, Mi was more prevalent in the normal group than in BC patients and, among these, was lower in ER+ PR− BC patients than ER− PR+ patients [12], thus suggesting that the protective role of Mi in BC development might be specific to ER + BC. These findings were only in part confirmed by the study by Winter et al., based on the Women’s Health Study cohort of 39,696 patients, which did not find an association between Mi and BC risk, but suggested an increased risk of HR-negative BC in women with a history of Mi [13].

A recent study, adding evidence to the role of Mi as a protective factor for BC, focused on combining the hormonal status of BC with the hormonal pattern of Mi, analyzing menstrual-related and menstrual-independent Mi separately [21]. In fact, menstrual Mi and menstrual-related Mi types are thought to be strongly influenced by fluctuations in estrogen levels throughout the menstrual cycle [22]. In this study, women with menstrual-related Mi showed a risk of developing HR-negative (ER−/PR−) cancer by about 40% less than women with non-menstrual-related Mi (hazard ratio = 0.63; 95% CI: 0.42–0.96; *p* = 0.005) [19].

Findings from epidemiological studies are summarized in Table 1.

## 4. Possible Causes for the Discrepancies among Studies Targeting the Associations between Mi and BC

One potential confounding factor when interpreting these large epidemiological studies is the diagnostic method applied to identify Mi. Headache differential diagnosis is based on the International Classification of Headache Disorders (ICHD-3), which is constantly updated according to new clinical and pathophysiological evidence and is currently in its third version (2018) [6]. However, among the cohort studies presented and included in this review, only the study of Ghorbani used ICHD to obtain a headache diagnosis, although they did not specify which version they used [12]. According to the publication date, the second version (2004) is the most likely to have been used.

Another point of interest is the temporal correlation between the two conditions. Mi is a fluctuating disorder that generally appears during adolescence and tends to disappear spontaneously with older age. Moreover, within the same individual and during every single year, Mi can range from an episodic to a chronic form [23] and possibly disappear for long intervals of time [24]. Considering these fluctuations, the influence exerted by Mi on BC might be different in cases where Mi afflicted patients for a limited period, many years before the BC diagnosis.

In two exploratory studies conducted by our group, ICHD criteria were used to obtain a headache diagnosis, and the status of activity of Mi (i.e., at least one Mi headache in the previous 12 months) was recorded at the moment of recruitment along with BC pathological data [25,26]. These studies provided results that only partially supported the investigations favoring a beneficial effect of Mi on BC, as our results suggested a possible co-existence of Mi and BC rather than a net beneficial effect. In fact, we found an overall high prevalence of Mi in our sample [25,26]. In addition, Mi patients showed a higher risk of having a stage II or III BC than stage I, and patients suffering from Mi (particularly Mi with aura (MwA)) showed an overall earlier onset of BC. Consistent with the hypothesis of an involvement of hormonal pathways in the relationship between Mi and BC, a positive correlation was observed among the frequency of headache attacks and the expression of ER and PR (i.e., the higher the expression of HR in BC, the higher the headache frequency). Specifically, BC patients with MwoA exhibited higher headache frequency in conjunction with higher expression of HR [26].

The present review of the current literature and all the meta-analyses revealed a relevant heterogeneity among different studies. These discrepancies can be attributed not only to methodological differences but also to a different association of Mi phenotypes and BC subtypes. The role of patient-related factors, including ethnicity and various environmental exposures, may further complicate the picture.

To improve future research, reviewing potential mechanisms underlying the interaction between Mi and BC would be valuable, thus designing studies on specific populations of Mi–BC patients based on their histological, instrumental, and clinical characteristics.

## 5. Female Reproductive Hormones

### 5.1. Hormonal Cycle Patterns

These epidemiological studies emphasize the significant role of female reproductive hormones and their receptors in the potential connection between BC and Mi. Specific patterns of hormonal variations are associated with both BC and Mi. MwA is associated with the peak of estrogen concentration, while MwoA is associated with rapid drops in estrogen concentration during the hormonal cycle [27]. On the other hand, BC is linked to high cumulative exposure to estrogens [28].

Notable differences have been highlighted between MwA and MwoA, regarding the posibility of experiencing headaches during menstruation, with a higher preference for MwoA [7,29]. It has been reported that a drop in circulating estrogen occurring 2–3 days before the onset of menses partially triggers menstrual-associated Mi [30]. Specifically, it has been found that 60% of women with Mi are more likely to have Mi attacks during peri-menstrual time periods and that there is a strong correlation between the onset of Mi (occurring between days 22 and 27) and the second dramatic drop of estrogens, which occurs in the luteal phase of the menstrual cycle. Similar findings had already been obtained in 1972 by Somerville and colleagues [31]. They demonstrated that women with Mi, who received a 10 mg intramuscular injection of estradiol, experienced a delayed Mi attack until the estradiol level returned to pre-treatment levels. In line with these data, epidemiologic findings showed that women suffer up to three times more from Mi in the peak of their reproductive years (20–40) compared with men, confirming the role of the hormonal cycle in headache prevalence. In more recent years, a multisite, multiethnic, longitudinal study has shown that female migraineurs are characterized by a faster decline in late luteal phase conjugated urinary estrogens (E1c) than controls [32]. Specifically, there were significant differences in E1c decline in the late luteal phase, and migraineurs showed a greater rate of E1c decline over the 2 days following the luteal peak than controls (40% vs. 30%; *p* < 0.001).

In contrast, women with MwA may experience more frequent attacks during estrogen peaks or during pregnancy [27]. On the other hand, the role of progesterone in Mi attacks appears to be less significant compared to estrogens [27], except possibly in relation to the intensity of the attack [33,34]. Its action appears to rely on reducing nociceptive inputs at the level of the trigeminal nucleus and dorsal horns directly and through its metabolite, allopregnanolone, which can modulate GABAA (γ-aminobutyric acid type A) receptors [35,36,37].

Other hormones, such as vasopressin, prolactin, and orexin, do not have a clear role in Mi [27]. Therefore, further studies in this field are needed. In addition, sex hormone-binding globulin (SHBG) has been investigated in Mi, demonstrating no association between SHBG and Mi [38]. However, it has been found that preventive antimigraine treatment with valproate could increase the serum level of SHBG in post-menarchal women [39].

Over the past 40 years, many studies have demonstrated a predominant role of estrogen exposure, both endogenous and exogenous, in BC development [28]. Endogenous exposure is closely linked to hormonal cycle patterns. In this context, in two Italian hospital-based case–control studies, the authors found that irregular menstrual patterns have a protective role in BC development [40,41]. Specifically, taking advantage of the large number of women enrolled in these studies (i.e., 1207 and 5606, respectively), the authors demonstrated that irregular menstrual patterns inversely correlate with the risk of both benign breast lesions (RR = 0.6; 95% CI: 0.4–1.0; *p* = 0.05 [40]) and BC development (RR = 0.4; 95% CI: 0.3–0.8; *p* < 0.05; and RR = 0.6; 95% CI: 0.5–0.8; *p* < 0.05, respectively; [40,41]). Similarly, breastfeeding has been shown to reduce BC risk [28], possibly by suppressing ovulation and consequently reducing exposure to estrogen. Other endogenous exposure factors that have been demonstrated to impact BC risk include the age at menarche and menopause. As extensively discussed in the 2003 review by Travis and Key [28], a 1-year delay in the onset of menarche was associated with a 5% reduction in BC risk [42]. Instead, each 1-year delay in the onset of menopause increased the risk by 3% [43]. In addition, it has been demonstrated that each birth reduces the BC RR by 7% [44]. To explain this protective mechanism, it has been suggested that the high serum levels of estrogens and progesterone during pregnancy stimulate the growth of the mammary epithelium and promote the differentiation of epithelial tissue, reducing the number of epithelial structures most vulnerable to epithelial transformations.

On the other hand, exogenous exposure to estrogens comprises the use of oral contraception and hormonal replacement therapy. Data from 54 published studies showed that the current or recent use (in the past 10 years) of oral contraceptives posed a slight increase in the BC RR [45]. However, further research is needed, as the formulations of oral contraceptives are changing. The use of hormonal replacement therapy showed a 2.3% risk of being diagnosed with BC for each year of use in the span of 1–4 years. Instead, the excess risk of being diagnosed with BC for patients who were on hormonal replacement therapy for 5 years or more was 35% [43]. Considering the type of hormonal replacement therapy, recent studies showed that using treatments containing estrogens plus progestins for 5 years induced a 26–30% increase in BC risk [46].

The role of estrogens in BC development was also confirmed by the demonstration that increased concentrations of circulating estrogens were linked to an increased risk of BC development in postmenopausal women. In fact, in a pooled analysis of nine prospective studies, including 663 postmenopausal BC patients, it was shown that the increase in circulating levels of estradiol, free estradiol, and oestrone significantly increased the risk of BC [47]. Circulating estrogen levels depended in part on the concentrations of SHBG. With high SHBG concentrations, the level of free circulating estrogen was lower; however, in obese postmenopausal women, the levels of SHBG were lower, thus increasing the exposure to free circulating estrogens [48].

We were not able to exclude the contribution of other hormones, such as progesterone, prolactin, and testosterone, in BC development, since current epidemiological and experimental data suggest a role for these hormones in the etiology of BC. Indeed, breast cell proliferation has been found to be greater during the luteal phase of the menstrual cycle, concomitant with high levels of progesterone [49]. In addition, high levels of prolactin have also been shown to increase BC risk because of the effect of prolactin in stimulating the proliferation, survival, and motility of mammary epithelial cells [50]. The conversion of testosterone into estrogen in the breast has been investigated in a pooled analysis of prospective studies, showing an increased BC risk in postmenopausal women with high testosterone levels [47].

### 5.2. The Association between Polymorphisms in Estrogen Synthesis Pathways and Mi and BC

Besides the hormonal cycle, changes in estrogen levels and their consequent effects on susceptibility to Mi and BC could also be due to mutations in estrogen pathways. The cytochrome p450 family 19 gene (*CYP19A1*), located on chromosome 15, encodes for the enzyme aromatase. Aromatase catalyzes the final step in estrogen biosynthesis and metabolism, converting androgens (androstenedione and testosterone) to estrogens (estrone and estradiol, respectively) [51]. Therefore, genetic alterations in this gene may affect estrogen synthesis and, in turn, influence the risk of Mi, BC, or both.

In this context, several polymorphisms have been evaluated in association with Mi or BC [52,53,54,55,56]. However, only the *CYP19A1* rs4646 (NC_000015.10:51210646:A:C, NC_000015.10:51210646:A:G in the gene *CYP19A1*) variant has been analyzed in both diseases. Specifically, in 2012 Ghosh and colleagues investigated the role of polymorphisms in *CYP19A1* and *ER* genes in the susceptibility to Mi in the North Indian population by conducting a case–control study and comparing it with other studies in a pooled meta-analysis [52]. Through genotyping experiments, the authors found that the *CYP19A1* rs4646 variant alone or in combination with *ESR1* mutations at locus rs9340799 (NC_000006.12:151842245:A:G, in the same gene) conferred a significant protective effect. In the context of BC risk, in 2015, a population-based case–control study conducted by Alanazi and colleagues explored the possible influence of *CYP19A1* polymorphisms on BC incidence in Saudi Arabian patients [53]. The genotyping analyses revealed no statistically relevant correlation between single nucleotide polymorphisms (SNPs) rs4646 and BC risk. Similarly, a previous investigation conducted in 2014 by Boone and colleagues showed an inverse correlation between *CYP19A1* rs4646 polymorphism and BC risk; however, after multiple comparison adjustments, no statistical significance was reached [54].

### 5.3. The Association between Polymorphisms in Estrogen Catabolism Pathways and Mi and BC

To be eliminated from the body, estrogens must be converted to inactive metabolites and then excreted in urine/feces. Estrogen metabolism is primarily dependent on cytochromes P450 (CYP450) enzymes (including *CYP1A1*, *CYP1A2*, *CYP1B1*, and *CYP3A4*), which are responsible for oxidation; UDP-glucuronosyltransferase, which manages glucuronidation; sulfotransferase, which induces sulfation; and catechol O-methyltransferase (*COMT*), which is responsible for O-methylation [57]. Hence, polymorphisms in genes related to the estrogen catabolism pathway may theoretically affect estrogen levels and, in turn, impact Mi and/or BC development.

However, despite preliminary in vitro findings suggesting that the mutant *COMT*-Met isoforms may increase BC risk due to variations in *COMT* catalytic activity associated with significant differences in the level of catechol estrogens [58], the role of *COMT* polymorphisms in BC risk has not yet been established, as results of several studies have been inconclusive or controversial [59,60]. Their investigations hypothesized that carrying these variants would increase BC susceptibility, as the *CYP1B1* variants displayed higher catalytic activity than the wild type [61]. In comparison, the *COMT* variants showed lower thermal stability and thus lower enzymatic activity, overall increasing the amount of “carcinogenic catechol estrogens”. However, neither the case–control study nor the meta-analysis showed any statistically significant association between BC risk and the *COMT* Val158Met (i.e., a non-synonymous G→A SNP (rs4680, NC_000022.11:19963747:G:A) in exon four leads to a valine (Val) to methionine (Met) peptide change in the mature protein) [60]. Finally, the lack of correlation between *COMT* Val158Met variants and BC development was confirmed in a large meta-analysis involving a total of 56 case–control observational studies conducted in 2012 by Qin and colleagues [62].

Similarly, no statistically significant association was found between *COMT* polymorphisms and Mi susceptibility. In fact, in a 2012 systematic review and meta-analysis regarding *COMT* polymorphisms and chronic pain [63], the authors did not find any correlation between the analyzed *COMT* SNPs and Mi. Specifically, the Val158Met polymorphism (rs4680, NC_000022.11:19963747:G:A), which has previously been shown to produce an enzyme with lower thermostability, thus causing a decrease in enzyme activity, was investigated in correlation with Mi (with and without aura). None of the three studies included in the analysis found any correlation between the aforementioned SNP and Mi. This lack of correlation was also observed in a case–control study exploring the possible association between four functional polymorphisms involved in estrogen metabolism and menstrual Mi in a UK population [64].

Besides *COMT* SNPs, *CYP1B1* polymorphisms have also been extensively investigated in association with BC [59,60,65,66]. The hypothesis behind these investigations is that *CYP1B1* may play a key role in breast and endometrial carcinogenesis [57]. The main activity of *CYP1B1* is represented by the 4-hydroxylation of estradiol, which in turn produces free radicals that may induce DNA damage. In addition, abundant levels of *CYP1B1* have been found in the so-called “estrogen target tissues”, which are mammary, ovarian, and uterine tissues, as well as in tumor tissues, thus suggesting that the specific and local 4-hydroxylation of estradiol could enhance carcinogenesis in these tissues [57]. However, the results of two meta-analyses highlighted the possible race-specific effects of *CYP1B1* polymorphisms [65,66]. Specifically, in the meta-analysis by Paracchini and colleagues, a lack of association between the *CYP1B1* Val432Leu (i.e., C→G transversion at position 1666 in exon 3, resulting in an amino acid substitution of leucine (Leu) with valine (Val) at codon 432; rs1056836, NC_000002.12:38071059:G:C) polymorphism and BC was observed in Asian individuals, whereas in populations of mixed/African origin, a negative correlation emerged (OR = 0.8; 95% CI: 0.7–0.9; *p* < 0.05). In addition, the pooled analysis showed a possible association in Caucasians (OR = 1.5; 95% CI: 1.1–2.1; *p* = 0.05), but this was age-dependent, being higher for the middle age classes (45–59 years) and lower among older and younger women. Unfortunately, no data on *CYP1B1* polymorphisms and Mi susceptibility have emerged so far.

### 5.4. The Association between Polymorphisms in ER Genes and Mi and BC

The *ESR1* gene is located on chromosome 6 and encodes for ER and ligand-activated transcription factor. The receptor plays a key role in BC, endometrial cancer, and osteoporosis. At the same time, the encoded protein has been found to play a role in growth, metabolism, sexual development, gestation, and other reproductive functions, via the regulating the transcription of many estrogen-inducible genes. Besides its well-known role in BC cancer management, the discovery that *ESR1* was localized in brain regions considered to be involved in Mi pathogenesis [67] suggested the role of ER in Mi development as well. The estrogen receptor α (ERα), related to the *ESR1* gene, and the estrogen receptor β (ERβ), related to the *ESR2* gene, activate a mitogen-activated protein kinase pathway [68,69]. In contrast, a third ER (G protein-coupled estrogen receptor 1, GPER) is coupled with a G-protein pathway [70]. ERα and GPER are expressed in the brainstem pons, and ERα in the periaqueductal grey [71]. Moreover, ERα and ERβ are expressed in the cerebral cortex, possibly explaining why estrogens are able to stimulate MwA [72]. Additionally, all three ERs are expressed in the hypothalamus, which has been found to be a key structure in regulating the recurrence of Mi attacks [73]. In particular, ERβ is mostly concentrated in the supraoptic area and the paraventricular nucleus [74]. ERβ-knockout murine models showed an increased level of the calcitonin gene-related peptide (CGRP); therefore, Krause et al. recently suggested that a direct action of estrogens on their receptors was able to alter the CGRP signaling and the recurrence pattern of Mi attacks [27].

Based on this hypothesis, polymorphisms in *ESR1* could represent a key point for understanding the relationship between BC and Mi susceptibility. Among the *ESR1* polymorphisms analyzed in correlation with both BC and Mi risk, one of the most investigated was an intronic polymorphism in the *ESR1* gene (rs2234693, NG_008493.2:g.190510T>C), also called *ESR1* PvuII. In the 2012 case–control study by Ghosh and colleagues, this intronic polymorphism was related to patients who had MwA [52]. On the contrary, no statistically significant correlations were observed between the *ESR1* PvuII polymorphism and the risk of any type of Mi in two different meta-analyses, each including eight studies [75,76].

The *ESR1* PvuII polymorphism (rs2234693, NG_008493.2:g.190510T>C) has been extensively evaluated in correlation with BC risk, with controversial results. In a cohort study published in 2008 by González-Zuloeta Ladd and colleagues [77], the authors found no correlation between the PvuII and BC risk in their Caucasian population. Similarly, a very recent retrospective case–control study showed that this variant was not associated with BC risk in Saudi women [78]. In contrast, in a previous large population-based cohort study, the authors showed that the PvuII pp genotype, compared with the PP one (CC-genotype), was a 1.5-fold increased BC risk. However, the correlation did not reach the threshold for statistical significance [79]. Interestingly, the authors also found a statistically significant interaction between PvuII polymorphism and E2 level on BC risk, and the effect was stronger among women with the Pp or pp genotypes. Consistently with these findings, in a large meta-analysis of 25 case–control studies, the authors showed that people with PvuII T > T + T > C or T > T genotypes were at a greater risk of BC than those with the C > C variant; however, regarding T > T polymorphism, the higher risk occurred only in an Asian population [80]. By contrast, in BC patients in Moskow, a significant association was found between the CC variant of the rs2234693 (NC_000006.12:151842199:T:C, NC_000006.12:151842199:T:G) polymorphism and the risk of BC development [81].

In the meta-analyses mentioned above by Li and Schürks [75,76], besides PvuII variants, the authors also analyzed *ESR1* 594 G > A and *ESR1* 325 C > G polymorphisms associated with Mi. In both studies, *ESR1* 594 G > A (rs2228480, NC_000006.12:152098959:G:A, NC_000006.12:152098959:G:T) and *ESR1* 325 C > G (rs2295190, NC_000006.12:152122608:G:T) polymorphisms were associated with Mi susceptibility, in a race-specific manner. In addition, in Schürks’s meta-analysis [76], both polymorphisms were associated with Mi, regardless of the presence of aura.

Given the BC risk, in 2012, an extensive review regarding ER gene mutations and polymorphisms in disease susceptibility attempted to bring together all the studies on this topic. It was found that the *ESR1* rs2228480 polymorphism was the most investigated among *ESR1* variants in correlation with BC risk [82]. Specifically, this variant was associated with BC risk in a considerable number of populations (e.g., Caucasian, Turkish, cohorts with Western European ancestries, Tunisian, and Korean).

## 6. Conclusions

This literature review aims to find the pathophysiological basis of the interconnection between Mi and BC susceptibility. On an epidemiological level, a protective effect has been found only in case–control studies but not in cohort ones and is not yet fully demonstrated. The inconsistency among different studies may be due to the method used to diagnose Mi and the age gap between the development of these two diseases but may also be due to confounding factors. For instance, it could be speculated that the use of preventive and acute medication for Mi may modify the subsequent risk of developing BC; however, this has not been confirmed by epidemiological evidence [17]. In addition, since Mi attacks can be triggered by alcohol consumption or smoking, two known risk factors for BC, some authors have hypothesized that migraineurs will avoid these habits [83,84]. While some studies have suggested this relationship, it has not been confirmed by other sources in which smoking and drinking habits were not different among patients with and without Mi [85,86,87].

According to epidemiological studies, HR-positive BC patients appeared to be the most influenced by Mi, as hormonal patterns and estrogen exposure were found to be strongly related to both disorders. One possible explanation regards the role of female reproductive hormones, exerting their action in both Mi and BC. In fact, hormonal patterns and estrogen exposure were shown to be strongly related to both disorders.

Analyzing the alterations in estrogen-related pathways, the relationship appears very complex when considering the number of SNPs and mutations that might be involved (Table 2). To date, the most promising alteration implicated in both Mi and BC has been found to involve the *ER gene α* and, specifically, the polymorphism *ESR1* 594 G > A (rs2228480, NC_000006.12:152098959:G:A, NC_000006.12:152098959:G:T). The polymorphism is linked to both Mi susceptibility and BC development, although further studies are definitely needed to investigate and validate this hypothesis. More interestingly, this polymorphism in Mi might be selectively associated with MwA more than MwoA, although no definitive evidence has been reached to date. This is food for thought in that MwA directly correlates with estrogens, i.e., the higher the estrogen levels, the higher the chance of experiencing MwA attacks, possibly through direct induction of the cortical spreading depression via ERs expressed on the cerebral surface. However, recent evidence has shown a direct correlation between the number of headache days and the level of ER and PR found in BC: specifically, the higher the expression of ER and PR, the higher the number of headache days in MwoA [26]. Therefore, we believe that indicating a preferential link between BC and MwA is currently too premature. We believe that further clinical studies should be conducted with a more rigorous evaluation of both BC and Mi diagnosis, in parallel with hypothesis-driven preclinical studies, in order to reduce study bias and to clearly understand the relationship between Mi and BC.

## Figures and Tables

**Table 1:** Summary of the epidemiological studies.

Authors	Title	Year	Type of Study	N. of Participants	Results	Protective Role of Mi on BC
Mathes, R.W., et al. [10]	Migraine in postmenopausal women and the risk of invasive breast cancer	2008	Combined data from two population-based case–control	1199 ductal BC 739 lobular BC 1474 controls	Postmenopausal women presenting a history of Mi had a reduced BC risk, in particular for HR + ductal (OR = 0.67; 95% CI: 0.54–0.82; *p* < 0.05) and lobular (OR = 0.68; 95% CI: 0.52–0.90; *p* < 0.05) BC.	Yes
Li, C.I., et al. [11]	Relationship between migraine history and breast cancer risk among premenopausal and postmenopausal women	2009	Population-based case–control study	4568 BC 4678 controls	Women with a history of Mi had a reduced risk of BC development (OR = 0.74; 95% CI: 0.66–0.82; *p* < 0.05), independent from menopausal status, age at diagnosis, use of Mi medications, and ability to avoid Mi triggers.	Yes
Ghorbani, A., et al. [12]	Evaluation of the relationship between breast cancer and migraine	2015	Case–control study	400 BC 400 controls	The frequency of Mi and TTH was more prevalent in controls than in BC women (OR = 2.54; 95% CI: 1.78–3.60; *p* < 0.001; and OR = 2.18; 95% CI: 1.51–3.15; *p* < 0.001, respectively).	Yes
Winter, A.C., et al. [13]	Migraine and subsequent risk of breast cancer: a prospective cohort study	2013	Cohort study	39,696 participants	Mi was not associated with BC risk (either total, in situ, or invasive BC). However, an increase in BC risk was shown in patients with an active diagnosis of MwoA at the time of the study (hazard ratio = 1.21; 95% CI: 1.03–1.43; *p* < 0.05).	No
Winter, A.C., et al. [14]	Migraine and breast cancer risk: a prospective cohort study and meta-analysis	2014	Cohort study + meta-analysis	115,378 participants for cohort study + 11,207 BC 10,818 controls (three case–control studies) 164,190 participants (three cohort studies) for meta-analysis	In the cohort study, Mi was not associated with BC risk (either total, in situ, or invasive BC). In the meta-analysis, an inverse association between Mi and BC risk was shown (pool RR = 0.84; 95% CI: 0.73–0.98; *p* ≤ 0.001), but the association was present only in case–control studies.	Only in case–control studies
Fan, C.Y., et al. [15]	Association between Migraine and Breast Cancer Risk: A Population-Based Cohort Study and Literature Review	2018	Population-based cohort study	25,606 Mi women 102,424 controls (frequency matched 1:4)	Mi was not associated with overall BC risk among Taiwanese women. However, the authors found a positive association between the risk of BC and the annual number of visits for Mi (hazard ratio = 2.00; 95% CI: 1.17–3.41; *p* = 0.0112) and between the presence of Mi and the number of BC-related examinations (*p* < 0.0001).	No
Rezaeian, S., et al. [16]	Migraine History and Breast Cancer Risk: A Systematic Review and Meta-Analysis	2015	Meta-analysis	7568 BC 6828 controls (four case–control studies) + 130,812 participants (two cohort studies)	Having any Mi history was inversely associated with BC risk (OR = 0.77; 95% CI: 0.64–0.92; *p* = 0.005). The protective effect persisted even when the analyses were stratified for age at diagnosis, histological type, or HR status.	Yes
Wu, X., et al. [17]	Migraine and breast cancer risk: a meta-analysis of observational studies based on MOOSE compliant	2016	Meta-analysis	7568 BC 6828 controls (four case–control studies) + 246,190 participants (three cohort studies)	A statistically significant inverse relationship between Mi and overall BC risk was found (RR = 0.78; 95%CI: 0.66–0.92; *p* < 0.05). However, the correlation seemed to be study-dependent, detected only in case–control studies and not in cohort ones.	Only in case–control studies
Hesari, E., et al. [18]	The association between migraine and breast cancer risk: A systematic review and meta-analysis	2022	Meta-analysis	12,322 BC 11,594 controls (five case–control studies) 426,493 participants (five cohort studies)	The authors found a statistically significant negative correlation between Mi and BC only in case–control studies (RR = 0.68; 95% CI: 0.56–0.82; *p* = 0.000) and not in cohort ones. Mi also reduced the risk of ductal and lobular carcinomas.	Only in case–control studies
Li, C.I., et al. [19]	Migraine history and breast cancer risk among postmenopausal women	2010	Cohort study	91,116 participants	Women with a Mi history showed a lower risk of BC development than those without Mi history (multivariate-adjusted hazard ratio = 0.89; 95% CI: 0.80–0.98; *p* < 0.05), independently from the use of non-steroidal anti-inflammatory drugs, and specifically in HR+ BC.	Yes
Lowry, S.J., et al. [20]	The risk of breast cancer is associated with specific patterns of migraine history; *Cancer Causes and Control*	2014	Population-based case–control study	715 BC 376 controls	Women with a long history of Mi (RR = 0.4; 95% CI: 0.2–0.6; *p* < 0.05), those who experienced MwA (RR = 0.7; 95% CI: 0.5–0.98; *p* < 0.05), and those who had their first Mi before age 20 (RR = 0.5; 95% CI: 0.3–0.9; *p* < 0.05) had a lower risk of ER+ ductal BC. Women with a long history of Mi (RR = 0.5; 95% CI: 0.3–0.9; *p* < 0.05), those who experienced MwA (RR = 0.6; 95% CI: 0.4–0.9; *p* < 0.05), and those who had their first Mi before age 20 (RR = 0.5; 95% CI: 0.3–0.9; *p* < 0.05) showed a lower risk of ER + lobular BC, too.	Yes
Shi, M., et al. [21]	Migraine and possible etiologic heterogeneity for hormone-receptor-negative breast cancer	2015	Case-control study (using two different cohorts)	50,884 women with a first-degree family history of BC 1418 case sisters who had been diagnosed recently with BC	Women with menstrual-related Mi showed a lower risk of developing an HR-BC than women with Mi independent from menses (hazard ratio = 0.63; 95% CI: 0.42–0.96; *p* = 0.005). The authors also found an inverse association between Mi and ductal carcinoma in situ.	Yes

Abbreviations: Mi, migraine; BC, breast cancer; HR, hormone receptor; ER, estrogen receptor; TTH, tension-type headache; MwoA, migraine without aura; MwA, migraine with aura; OR, odds ratio; CI, confidence interval; RR, relative risk.

**Table 2:** Main results on SNPs in estrogen pathways and Mi susceptibility and BC risk.

Pathway	SNP	Mi Susceptibility	BC Risk	Ref
Estrogen synthesis	*CYP19A1* rs4646	↓	-	[52,53,54]
Estrogen catabolism	*COMT* rs4680	-	-	[60,62,63,64]
	*CYP1B1* rs1056836	n.d.	↓	[66]
Estrogen receptor genes	*ESR1* rs2234693	↑-	↑↓-	[52,75,76,77,78,79,80,81]
	*ESR1* rs2228480	↑	↓	[75,76,82,88]

Abbreviations: Mi, migraine; BC, breast cancer; *CYP19*, cytochrome p450 family 19 gene; *ESR1*, estrogen receptor 1; *COMT*, catechol O-methyltransferase; SNP, single nucleotide polymorphism; ↓, decreased; ↑, increased; -, no statistically significant association; n.d., no data; Ref, reference.

## Data Availability

Not applicable.

## Supplementary Materials

The following supporting information can be downloaded at: https://www.mdpi.com/article/10.3390/biomedicines11061613/s1, Table S1: Association of hormone replacement therapy with BC risk and Mi incidence.
