# Supplementary material for: The Role of Female Reproductive Hormones in the Association between Migraine and Breast Cancer: An Unanswered Question
Source: Biomedicines. 2023 Jun 1;11(6):1613. doi: 10.3390/biomedicines11061613 (PMC10295631; doi:10.3390/biomedicines11061613)
Supplement: Supplementary file 1 [file biomedicines-11-01613-s001.zip › biomedicines-2396672-supplementary.pdf]

Supplementary Materials

Table S1. Association of hormone replacement therapy with BC risk and Mi incidence.

HRT Type	BC risk RR (95% CI) [5]	Mi incidence OR (95% CI) [9]
Estrogen only	1.30 (1.22–1.38)	1.70 (1.50– 1.92)
Estrogen–progestin	2.00 (1.91–2.09)	1.42 (1.25– 1.61)

Abbreviations: HRT, hormone replacement therapy; BC, breast cancer; Mi, migraine; RR, relative risk; OR, odds ratio; CI, confidence interval.
